# Supplementary material for: Content-rich biological network constructed by mining PubMed abstracts
Source: BMC Bioinformatics. 2004 Oct 8;5:147. doi: 10.1186/1471-2105-5-147 (PMC528731; doi:10.1186/1471-2105-5-147)
Supplement: Additional File 2 — The original results of the above study (non-essential files are deleted to keep the file size under the limit set by BMC bioinformatics). [file 1471-2105-5-147-S2.bz2 › chilibotAdditionalFile2/dip10/697ID11585365E18023/html/MUTL_VSR.html]

 


 **MUTL** and **VSR** 
  
Found 10 abstracts in PubMed, retrieved 10.  
 

 What does Google say? 
 PDF only 
| .edu only 

---

**Interactive relationship** (e.g. stimulation, inhibition, etc)

**Stimulatory relationship**- The Escherichia coli  **MutL**  protein stimulates binding of  **Vsr**  and MutS to heteroduplex DNA.  Ref: 9461452 Nucleic Acids Res, 1998
- It depends on genes  **vsr**  and polA and is strongly stimulated by  **mutL**  and mutS.  Ref: 1944537 Nature, 1991
**Neutral relationship**- Purified  **MutL**  appears to act catalytically to bind  **Vsr**  to its substrate.  Ref: 9461452 Nucleic Acids Res, 1998
- This result is discussed in the light of previous in vitro and in vivo data which indicate that the  **MutL**  protein may be needed for full  **vsr**  activity.  Ref: 11124025 J Mol Biol, 2000
- Reduction in VSP repair caused by the lack of  **MutL**  or MutS was fully complemented by the addition of  **vsr**  plasmids when the T G mispair was in the 5 CTAGG 3 GGTCC context.  Ref: 7836300 J Bacteriol, 1995

**Non-interactive relationship** (e.g. studied together, co-existance, homology, etc.)

- In this study, we use a bacterial two hybrid assay to show that  **MutL**  interacts with  **Vsr** .  Ref: 11585365 Mutat Res, 2001
- We also show that interaction between  **Vsr**  and  **MutL**  inhibits the ability of  **MutL**  to dimerize, to interact with MutS and MutH and to mediate a previously unknown interaction between MutS and MutH.  Ref: 11585365 Mutat Res, 2001
